# Recent Developments in Chemo-Photodynamic Nanoplatforms for Glioblastoma Treatment

**DOI:** 10.3390/ijms27209058

**Published:** 2026-10-13

**Authors:** Paweł Woźnicki, David Aebisher

**Affiliations:** 1 Doctoral School, Faculty of Medicine, Collegium Medicum, University of Rzeszów, 35-310 Rzeszow, Poland; pawelw@dokt.ur.edu.pl; 2 Department of Photomedicine and Physical Chemistry, Faculty of Medicine, Collegium Medicum, University of Rzeszów, 35-310 Rzeszow, Poland

**Keywords:** nanoplatform, PDT, chemotherapy

## Abstract

Glioblastoma multiforme (GBM) remains one of the most difficult malignant brain tumours to treat due to its infiltrative nature, cellular heterogeneity, resistance to treatment and limited drug penetration across the blood–brain barrier. The combination of photodynamic therapy (PDT) with chemotherapy within a single nanoplatform offers the possibility of integrating the photogeneration of reactive oxygen species with the cytotoxic action of chemotherapeutic agents, whilst improving their coordinated delivery to tumour cells. This review summarises the latest nanoparticle-based strategies combining PDT and chemotherapy in the treatment of GBM. The literature published between January 2025 and August 2026 was identified using the PubMed, Web of Science and Scopus databases. The systems analysed included photoreactive organic nanoaggregates, ZnO-based nanocomposites, lipid nanocarriers, dual-targeting liposomes and platforms based on covalent organic frameworks. These nanoplatforms enabled various forms of therapeutic integration, including light-triggered drug release, co-encapsulation of photosensitisers and chemotherapeutic agents, targeting of mitochondria, and targeting of glioma cells and blood–brain barrier models via ligands. The available studies have generally demonstrated increased cytotoxicity following photoactivation, and selected systems have shown synergistic effects between chemotherapy and PDT. However, the available evidence is insufficient to support the translational readiness of the currently reported chemo-PDT nanoplatforms. Three of the five systems were evaluated only in vitro, only one was investigated in an orthotopic glioma model, and several studies relied on surrogate metabolic assays or irradiation conditions of limited relevance to deep intracranial disease. The current literature should therefore be regarded primarily as proof-of-concept evidence for coordinated chemo-PDT delivery rather than as evidence of clinically applicable therapy. Progress towards clinical relevance will require platforms designed around realistic light-delivery constraints, the hypoxic GBM microenvironment, brain-relevant delivery routes and therapeutically meaningful in vivo endpoints.

## 1. Introduction

Glioblastoma (GBM) is the most common primary malignant brain tumour in adults and, despite the use of multimodal treatment, continues to be associated with a very poor prognosis. Standard management involves maximal safe tumour resection, followed by radiotherapy in combination with temozolomide and subsequent adjuvant chemotherapy; however, the median survival remains less than two years, and the disease recurs in the majority of patients [1,2]. Effective treatment of GBM is hampered by its highly infiltrative nature, significant heterogeneity and cellular plasticity, the presence of resistant glioblastoma stem cell subpopulations, the immunosuppressive tumour microenvironment, and the blood–brain barrier, which limits the access of many therapeutic agents to the tumour tissue [1]. The complexity of these mechanisms suggests that effective treatment of GBM may require simultaneously targeting several different aspects of tumour biology, rather than focusing on a single therapeutic target [3].

One method with the potential to be incorporated into such strategies is PDT, which relies on the interaction of a photosensitiser, molecular oxygen and light at a wavelength that overlaps with the absorption spectrum of the photosensitiser, preferably within a spectral region that allows effective penetration through biological tissues. Activation of the photosensitiser leads to type I and/or type II photochemical reactions and the generation of reactive oxygen species (ROS), which induce oxidative stress and the death of cancer cells [4,5]. However, the mechanism of action of PDT is not limited to direct cytotoxicity, as it also involves damage to the tumour vasculature and the induction of a host immune response [5]. Particularly promising is PDT’s ability to induce immunogenic cell death, leading to the exposure and release of DAMPs and tumour antigens, and consequently to the activation of an anti-tumour response [6,7].

Despite these advantages, PDT used as a standalone therapeutic strategy has significant limitations. Hypoxia impairs the efficacy of oxygen-dependent reactions, and the further consumption of oxygen during PDT may exacerbate local hypoxia even further. The efficacy of the therapy is also limited by the poor penetration of light into the tissues, the non-uniform accumulation of the photosensitiser, its aggregation, and the possibility of non-specific distribution in healthy tissues [7,8]. These limitations also provide a rationale for combining PDT with methods that act via different, partly independent anti-tumour mechanisms.

One of the most obvious combination strategies is the combination of PDT with chemotherapy. Chemotherapeutic agents act on cancer cells via mechanisms distinct from the oxidative stress induced by PDT, including DNA damage or disruption of cell proliferation and survival processes [9,10]. Such a combination may enable simultaneous targeting of various pathways responsible for the survival and resistance of GBM cells, and in some cases may also lead to a synergistic effect [11]. Of particular importance is the ability to coordinate both components of the therapy in terms of both space and time, for example through the simultaneous administration of a photosensitiser and a cytotoxic drug, or the light-controlled release of a chemotherapeutic agent.

Nanotechnology may play a key role in implementing such strategies. Nanocarriers can improve the solubility and stability of photosensitisers, limit their aggregation, increase their accumulation in tumour tissue and enable more selective delivery to target cells [7,12]. Importantly, PDT and chemotherapy do not necessarily need to be incorporated into the same nanocarrier and may also be administered as separate therapeutic modalities. The rationale for integrating them within a single nanoplatform is therefore not to make the chemotherapeutic effect dependent on light, but rather to improve the spatial and temporal coordination of their delivery. When administered separately, a photosensitiser and a chemotherapeutic agent may differ in pharmacokinetics, tumour accumulation, cellular uptake and intracellular localisation, potentially reducing the likelihood that therapeutically effective concentrations of both agents reach the same tumour cells within the same time window. Co-delivery within a single nanoplatform may partly reduce this mismatch by enabling both components to share the same targeting and transport properties and, in selected systems, by allowing controlled or stimulus-responsive drug release. This may favour simultaneous exposure of tumour cells to photodynamic damage and chemotherapy and thereby facilitate synergistic interactions [13,14]. However, such integration is not intrinsically superior to separate administration. It may be disadvantageous when the two therapeutic components require different doses, pharmacokinetic profiles or treatment schedules, and it also increases formulation complexity. Moreover, chemotherapy may retain activity outside the irradiated region, whereas the photodynamic effect remains restricted to tissue receiving an effective light dose. Thus, a combined nanoplatform does not overcome the fundamental spatial limitation of PDT but may improve the coordination of both modalities within the illuminated tumour volume.

This review summarises the latest strategies based on nanoplatforms combining photodynamic therapy with chemotherapy in the treatment of glioblastoma. The literature published between January 2025 and August 2026 was searched in PubMed, Scopus and Web of Science using the Boolean query “PDT AND nanoparticles”. Original experimental studies were eligible if they directly investigated glioblastoma, involved photodynamic therapy or a directly PDT-related photosensitizer platform, and employed a nanoparticle, nanocarrier or other nanostructured therapeutic system. Non-original publications, studies unrelated to GBM, studies not involving PDT and studies without a nanostructured therapeutic platform were excluded. The search yielded 3617 records, of which 35 studies met the broader eligibility criteria for nanoparticle-mediated PDT in GBM. Among these, five additionally combined PDT with chemotherapy and constituted the complete set of studies included in the focused chemo-photodynamic analysis.

## 2. The Architecture of Nanoparticles

The nanoplatforms analysed exhibit considerable architectural diversity—ranging from single-component organic nanoaggregates and supramolecular inclusion complexes to porous, multi-component drug delivery systems and biohybrid platforms utilising living cells as carriers. The simplest structure is represented by Acr-Cbl nano, a single-component organic nanoplatform based on a π-extended acridine photocage; in this designation, “Acr” refers to the acridine-based photoactive moiety and “Cbl” to chlorambucil [15]. Unlike conventional nanocarrier systems, the photosensitiser and the drug are not incorporated separately into an additional carrier material in this case [16]. Photocatalysts are a class of substances containing light-sensitive groups, from which target substances are released when exposed to light of specific wavelengths [17]. In the Acr-Cbl system, the acridine chromophore was covalently linked to chlorambucil, forming a single conjugate that acts both as a photodynamic module and a source of the chemotherapeutic agent. The resulting compound was converted into nanoparticles using the reprecipitation method. In an aqueous environment, the hydrophobic particles formed globular nanoaggregates with an average diameter of approximately 112 nm, without the need for an additional polymer matrix or coating. Under the influence of visible light, however, the photolabile framework disintegrated via heterolytic cleavage of the C–O bond, enabling the release of active chlorambucil, whilst the acridine moiety was responsible for generating singlet oxygen [15].

In the NCP platform (ZnO-Cys-Flx-TMZ-MB), where the designation reflects its principal components—ZnO, cysteine, fluoxetine, temozolomide and methylene blue—a more targeted delivery strategy was employed. The core of the system consists of zinc oxide nanoparticles (ZnO NPs), which are used as a nanocarrier for the other components [18]. ZnO is a wide-bandgap oxide semiconductor exhibiting photoactive and photocatalytic properties. More broadly, ZnO belongs to the group of wide-bandgap semiconductors increasingly investigated for biomedical applications owing to their optical and electronic properties [19]. In its nanoparticle form, it is characterised by a high surface-to-volume ratio, which provides an extensive surface area available for the adsorption of compounds and chemical modification. The ability to functionalise the ZnO surface using ligands, polymers and other organic molecules allows for the design of multi-component drug delivery systems and the introduction of additional therapeutic functions. For this reason, ZnO NPs are increasingly being used as a component of multimodal anti-cancer nanoplatforms, in which they can combine the function of a nanocarrier with photoactive properties [20,21]. In the system under investigation, the ZnO NPs used had an average size of approximately 25 nm, and their surface was functionalised with L-cysteine (Cys) in the first stage [18]. Thanks to the presence of thiol, amine and carboxyl groups, cysteine can act as a linking molecule, enabling further functionalisation of the surfaces of inorganic nanoparticles [22]. Fluoxetine (Flx), temozolomide (TMZ) and methylene blue (MB) were then incorporated into the resulting system, creating a multifunctional composite containing a targeting ligand, a chemotherapeutic agent and a photosensitiser [18]. In this structure, ZnO therefore forms the inorganic scaffold of the nanoplatform, whilst the organic components confer additional biological and photoactive functions upon it.

Another group consists of lipid nanoplatforms, an example of which is Cela@Lip-IRH [23]. Liposomes and related lipid nanoparticles are among the most commonly used drug delivery systems due to their biocompatibility, their ability to encapsulate compounds with varying degrees of hydrophobicity, and the ease with which their surfaces can be modified [24,25,26]. Liposomes are based on amphiphilic phospholipids, which, in an aqueous environment, spontaneously form a lipid bilayer surrounding an aqueous, hydrophilic interior. The hydrophobic lipid chains, in turn, form the hydrophobic domain of the membrane, which enables the encapsulation of compounds with diverse physicochemical properties [27], in the case of Cela@Lip-IRH celastrol [23]. In Cela@Lip-IRH, the main lipid matrix consisted of lecithin and cholesterol, supplemented with DSPE-PEG derivatives [23]. Lecithin provides the phospholipids that form the bilayer structure of the carrier, whilst cholesterol is incorporated between their molecules, stabilising the lipid bilayer. This increases the orderliness of the bilayer, regulates its fluidity and permeability, and limits the premature loss of the encapsulated cargo [27]. Another important component of the architecture was the DSPE-PEG derivatives, whose hydrophilic chains can form a hydration layer on the surface of the nanoparticles, thereby limiting aggregation and the adsorption of serum proteins [28,29]. In the first stage, IR780 was covalently attached to DSPE-PEG-NH_2_, yielding DSPE-PEG-IR780, which was then incorporated into the structure of the lipid nanoparticles. In this configuration, the hydrophobic lipid segment of DSPE allows the conjugate to be anchored within the lipid bilayer, whilst the hydrophilic PEG chain extends towards the aqueous environment, enabling the attached IR780 to be exposed on the surface of the nanoplatform. IR780 served both as a photosensitiser and as the component responsible for mitochondrial targeting [23].

A more complex liposomal architecture was employed in the case of BTP-7/BR2-ICG/MTO-LP. The liposomes were prepared using the thin-film hydration method, incorporating the photosensitiser indocyanine green (ICG) and the chemotherapeutic agent mitoxantrone (MTO) into a single carrier. The structural basis of the nanoplatform was a bilayer composed of phosphatidylcholine (PC) and cholesterol [30]. Phosphatidylcholine is an amphiphilic glycerophospholipid composed of a hydrophilic phosphocholine group and two hydrophobic fatty acid chains. This structure facilitates its self-organisation in an aqueous environment and the formation of stable lipid bilayers; consequently, PC is one of the most commonly used basic components of liposomes. Furthermore, the length and degree of saturation of its acyl chains influence the packing, fluidity and permeability of the resulting membrane [31]. Cholesterol, by becoming embedded between phospholipid molecules, further regulates the organisation and stability of the bilayer [27]. Another key component of the structure was the two lipid-polymer conjugates, DSPE-PEG–BTP-7 and DSPE-PEG–BR2, which perform a targeting function and are introduced during the formation of the lipid layer [30]. As noted above, in structures of this type, the hydrophobic DSPE segment anchors the conjugate within the lipid bilayer, whilst the hydrophilic PEG chain faces the aqueous environment and allows the attached ligands to be exposed on the surface of the nanoparticle. The BTP-7/BR2-ICG/MTO-LP system thus represents an example of a multi-level architecture in which the co-encapsulation of two active compounds is combined with the simultaneous incorporation of two PEG-functionalised lipids into the surface layer of the nanocarrier.

**Figure 1:**
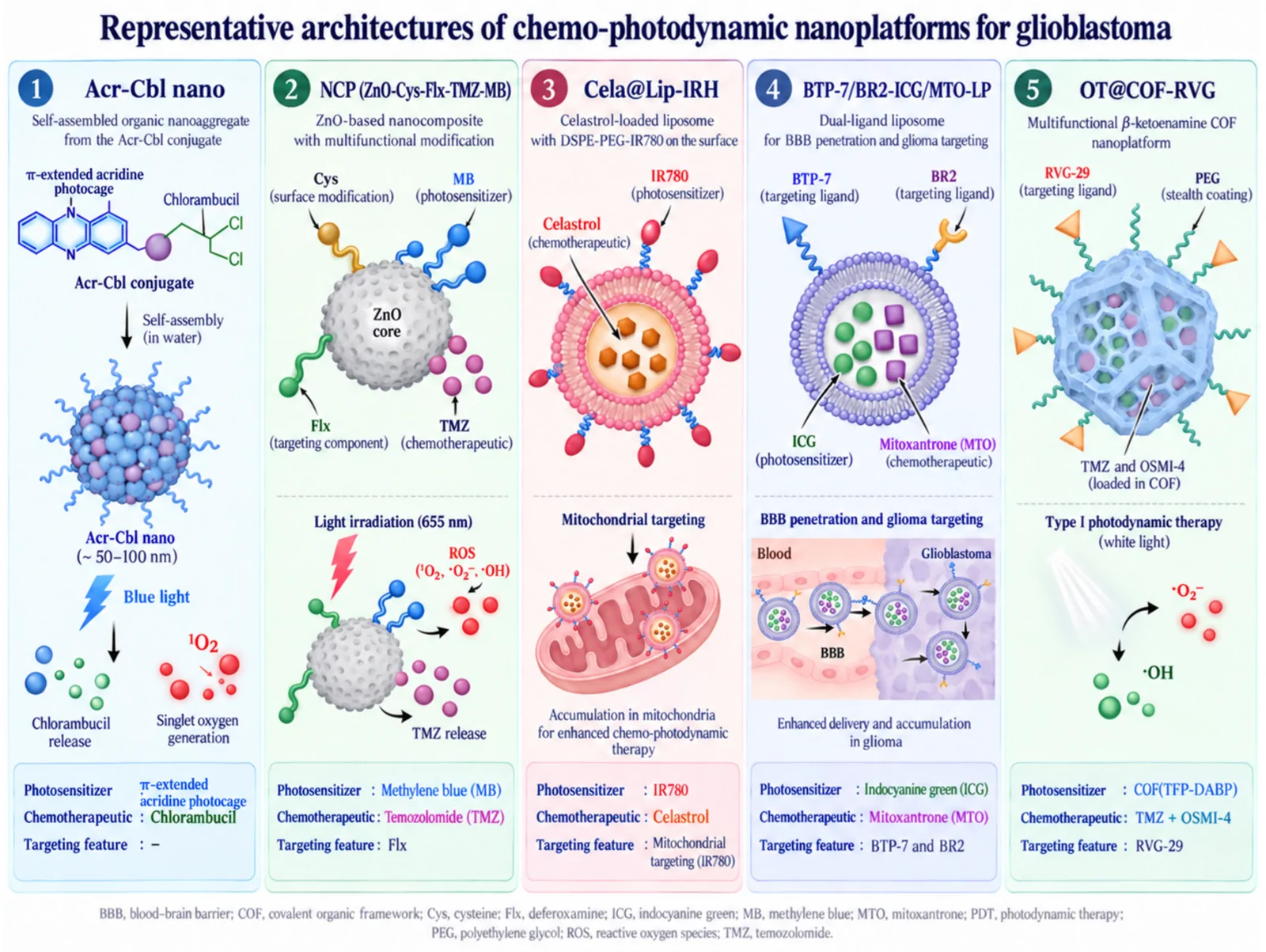
Representative architectures of chemo-photodynamic nanoplatforms for glioblastoma.

The most complex architecture in terms of material organisation is represented by OT@COF-RVG, which is based on a β-keto-enamine covalent organic network [32]. Covalent organic frameworks (COFs) are crystalline, porous materials composed of organic units linked by permanent covalent bonds. They are characterised by a high specific surface area, ordered porosity and the ability to precisely modify their composition and surface, making them attractive scaffolds for the encapsulation and co-delivery of therapeutic compounds [33,34]. In the case of OT@COF-RVG, the ordered COF_TFP-DABP_ network was formed through the covalent bonding of TFP and DABP units, which constitute a porous β-ketoamine scaffold. OT@COF-RVG was then prepared in three stages. Following the formation of COF_TFP-DABP_, temozolomide and an O-GlcNAc transferase inhibitor (OSMI-4) were simultaneously encapsulated within its pores, yielding OT@COF. In the final stage, the surface of the nanoparticles was coated with amino-functionalised PEG, and the RVG-29 targeting peptide was subsequently attached, resulting in the final OT@COF-RVG formulation [32]. OT@COF-RVG is therefore a hierarchically organised nanoplatform comprising a porous COF, two compounds embedded within its pores, and an outer layer of PEG functionalised with the RVG-29 peptide (Figure 1).

## 3. Photosensitisers Used

The nanoplatforms analysed utilise photoactive components belonging to various chemical classes, including acridine photocages, phenothiazine and cyanine dyes, and photoactive covalent organic networks. The irradiation conditions used in the analysed studies ranged from visible-light excitation (λ ≥ 410 nm for Acr-Cbl nano and broadband white light for OT@COF-RVG) to red-light irradiation at 655 nm and NIR irradiation at 808 nm.

In the Acr-Cbl nano, the photoactive function is performed by a π-extended acridine photocage covalently coupled to chlorambucil [15]. Acryidine is a heteroaromatic chromophore used in the design of photoactive biological systems, and the extension of the π system allows the absorption properties to be shifted towards the visible light spectrum [35,36]. Studies have shown that, upon irradiation with visible light transmitted above a 410 nm cut-off (λ ≥ 410 nm), Acr-Cbl nano releases chlorambucil and generates singlet oxygen (^1^O_2_). Based on the results obtained and data from the literature, the authors proposed a mechanism whereby the excitation of Acr-Cbl to the singlet state leads to heterolytic cleavage of the C–O bond and the release of chlorambucil, whilst the intersystem transition to the triplet state enables energy transfer to molecular oxygen and the formation of ^1^O_2_. Importantly, the photolysis product Acr-OH also retained the ability to generate ^1^O_2_. Acr-Cbl nano exhibited a high quantum yield for this process (ΦΔ = 0.58), higher than that of Acr-OH (ΦΔ = 0.49) [15]. A key advantage of Acr-Cbl is therefore the integration, within a single structure, of two light-dependent processes—the generation of ^1^O_2_ and the controlled release of the chemotherapeutic agent. A major limitation of Acr-Cbl, however, is its requirement for short-wavelength visible-light irradiation. Although the original study used a broad visible-light source with a 410 nm cut-off rather than monochromatic 410 nm irradiation, wavelengths near the lower end of this range undergo strong scattering and attenuation in tissue. In the 400–430 nm region, the reported optical penetration depth is below 0.5 mm [37]. This limitation is particularly relevant to GBM because tumour cells can infiltrate apparently normal brain tissue far beyond the macroscopic tumour margin; biopsy studies have detected malignant glioma cells at distances of up to approximately 4 cm from the visible lesion [38]. This value refers to the extent of tumour-cell infiltration, not to the penetration depth of activating light. Effective light delivery over such a distance would be negligible, particularly at short visible wavelengths. The marked disparity between the spatial extent of infiltrative disease and the limited penetration of activating light therefore represents an important limitation of Acr-Cbl nano for treating the diffuse component of GBM.

In the NCP nanocomposite, methylene blue (MB) acted as the photosensitiser [18]. MB is a cationic phenothiazine dye with intense absorption in the red region of the spectrum, with a maximum at approximately 660–665 nm, making it well suited for activation by red light used in PDT [39,40]. It is also characterised by a high molar extinction coefficient and a relatively high quantum yield for singlet oxygen generation [41]. Upon excitation, MB transitions to a triplet state, from which it primarily participates in type II reactions, leading to the generation of singlet oxygen (^1^O_2_) via energy transfer to molecular oxygen. Under certain conditions, a type I mechanism may also be involved, comprising electron transfer processes and the formation of radical forms of ROS, such as the superoxide anion radical, and, indirectly, H_2_O_2_ and the hydroxyl radical [39,40]. A well-known limitation of MB, however, is its tendency to aggregate and self-extinguish in an aqueous environment, particularly at higher concentrations. Aggregation may reduce the efficiency of energy transfer from the triplet state to oxygen, leading to a decrease in the efficiency of ^1^O_2_ generation [42]. In the nanoplatform under investigation, activation was carried out at 655 nm, and the incorporation of MB into the multicomponent nanocomposite significantly altered its optical properties. The extinction coefficient increased from 0.08 for MB to 3.21 mL mg^−1^ cm^−1^ for NCP, i.e., approximately 40-fold. The authors noted that the increased absorption at the laser wavelength was associated with increased ROS production by the NCP. An additional component of the NCP is ZnO nanoparticles with photocatalytic properties; however, MB remains the actual molecular photosensitiser activated at 655 nm [18].

In the case of Cela@Lip-IRH, IR780 was used, a lipophilic cationic dye from the cyanine group, which exhibits strong absorption in the near-infrared region [23]. The cyanine chromophore consists of two terminal heterocyclic units linked by a conjugated polymethine chain; the extensive π-bond network in heptametine cyanines shifts the absorption into the NIR range, typically around 725–850 nm [43]. IR780 exhibits a maximum absorption at approximately 780 nm [44]; as a result, it can be effectively activated by NIR radiation, which penetrates tissues more deeply than short-wavelength visible light [37]. However, the limitations of free IR780 remain its very low solubility in aqueous media and its limited photostability, which justifies its incorporation into nanocarrier systems [44]. Following the incorporation of IR780 into Cela@Lip-IRH, a shift in the absorption maximum from approximately 780 to 790 nm was observed, whilst activity in the NIR range was maintained [8]. The effective generation of ^1^O_2_ following irradiation with an 808 nm laser was also confirmed—after 180 s, the absorbance of DPBF had fallen to approximately 12.8 per cent of its initial value, whilst nanoparticles lacking IR780 showed no significant photosensitising activity. These results confirm that the photodynamic properties of IR780 are preserved following its incorporation into the nanoplatform [23]. IR780 also acts as a mitochondrial-targeting agent in this nanoplatform, owing to its ability to accumulate preferentially in these organelles; this property has been confirmed, amongst others, in T98G and U87MG glioma cells [45]. The significance of this mechanism for the subcellular targeting of the therapy is discussed later in this paper.

In BTP-7/BR2-ICG/MTO-LP, indocyanine green (ICG), a cyanine dye, acts as the photosensitiser [30]. ICG is an amphiphilic tricarbo-/heptametinocyanin dye with strong absorption in the near-infrared region. In an aqueous environment, the absorption maximum occurs at around 780 nm, but upon binding to proteins and in a biological environment, the absorption band may shift towards approximately 800–805 nm [46,47,48]. Once excited, ICG can lead to the formation of ROS and, at the same time, dissipate some of the absorbed energy as heat, thereby exhibiting both photodynamic and photothermal properties [49]. Despite its favourable absorption in the NIR region, ICG is characterised by a relatively low quantum yield for singlet oxygen generation, the value of which is highly dependent on the environment and measurement conditions [50]. Further limitations of free ICG include aggregation in aqueous solutions, photodegradation, and the dependence of its spectral properties on concentration and microenvironment [51,52], which is one of the reasons for its inclusion in nanocarriers [53]. In the ICG nanoplatform under investigation, ICG was activated by NIR light at a wavelength of 808 nm. Following irradiation, increased ROS generation was observed in C6 cells, confirming that ICG retains its photodynamic activity after incorporation into liposomes. At the same time, a pronounced photothermal effect was observed, indicating that ICG retains dual photo-induced activity within this platform, combining ROS generation with the conversion of light energy into heat [30].

A different approach was adopted in OT@COF-RVG, in which no conventional molecular photosensitiser was introduced; instead, the photoactive function is performed directly by the β-ketoenamine covalent organic framework COF_TFP-DABP_ [32]. Photoactive COFs can form complex, π-conjugated donor-acceptor systems, the electronic structure of which can be designed to increase charge separation and enhance ROS generation [54,55]. In COF_TFP-DABP_, the photodynamic properties are governed by a pH-dependent intramolecular proton transfer process in the excited state (ESIPT) and the equilibrium between the keto and enol forms [32]. ESIPT is a phenomenon occurring in photophysical processes, involving the transfer of protons from one location to another within a molecule. As with covalent organic structures based on β-ketoamides, this manifests as the transfer of a proton from a hydroxyl group to an adjacent nitrogen atom. This occurs particularly under acidic conditions, in which the nitrogen sites in the β-ketoamine structure undergo protonation [56]. Following irradiation with white light (26 mW cm^−2^), COF_TFP-DABP_ was shown to generate primarily the superoxide anion radical (O_2_•^−^) and the hydroxyl radical (•OH). The generation of both O_2_•^−^ and •OH was further confirmed by ESR, whereas no significant generation of singlet oxygen (^1^O_2_) was observed [32]. These results indicate that COF_TFP-DABP_ acts primarily via a type I PDT mechanism. This mechanism may be of particular significance in GBM, whose microenvironment is characterised by extensive hypoxia resulting, amongst other things, from abnormal vascularisation and insufficient tumour perfusion [57]. Limited oxygen availability poses a significant challenge for PDT, particularly for the type II mechanism, in which O_2_ is directly utilised to generate ^1^O_2_; furthermore, the photodynamic reaction itself may consume additional oxygen and exacerbate localised hypoxia. For this reason, the predominant type I mechanism, COF_TFP-DABP_, which is less dependent on oxygen availability and leads to the formation of radical ROS, may represent a beneficial property in the context of the hypoxic microenvironment of GBM [58,59,60].

Importantly, limited tissue penetration of activating light does not necessarily require illumination exclusively from the tissue surface. In interstitial PDT (iPDT), one or more optical diffuser fibres can be stereotactically positioned within the tumour, thereby delivering light directly to deeply located lesions and reducing the distance that photons must propagate through brain tissue. This approach has already been clinically investigated in GBM using 5-ALA-induced protoporphyrin IX and 635 nm light delivered through stereotactically implanted cylindrical diffuser fibres [61]. For surgically accessible tumours, intraoperative intracavitary illumination represents another strategy, in which a light-diffusing device is positioned within the resection cavity to irradiate the surrounding tumour bed and residual cells at the cavity margin [62]. However, neither approach fully resolves the problem of diffusely infiltrating GBM cells located at substantial distances from the treated volume. Their effectiveness therefore depends on appropriate fibre placement, treatment planning and light dosimetry, while the development of photosensitisers and nanoplatforms activated at longer wavelengths may further increase the tissue volume reached around each light source [63].

## 4. Chemotherapy Drugs Used

Combining photodynamic therapy with chemotherapy allows for the simultaneous utilisation of different mechanisms for damaging cancer cells. Chemotherapy drugs primarily target DNA or key cellular survival pathways, whilst PDT leads to the generation of ROS following the activation of the photosensitiser by light. The integration of both methods into a single nanoplatform may enhance the co-delivery of therapeutic agents to tumour cells and enable them to reinforce each other’s effects [64,65,66]. In the systems analysed, this was achieved through light-controlled drug release, the co-encapsulation of a photosensitiser and a chemotherapeutic agent, or by directing both mechanisms towards the same cellular compartment.

In Acr-Cbl nano, chlorambucil was used as the chemotherapeutic agent [15]. Chlorambucil is a bifunctional alkylating agent belonging to the nitrogen mustard group, whose anticancer effect results from covalent modification of DNA and the formation of cross-links, particularly involving guanine, which disrupts replication and leads to cell death [67]. Its activity against glioma cells has been demonstrated in several glioma cell lines in vitro [68,69]. Despite its alkylating activity, chlorambucil has not found widespread clinical use in the treatment of GBM, and the available clinical data are limited and derive mainly from older studies; in a phase II trial of prednimustine, a chlorambucil derivative, in 21 patients with GBM, a median survival of 8.7 months was achieved, whilst the efficacy of the treatment was assessed as limited [70]. In Acr-Cbl nano, chlorambucil was covalently linked to an acridine photosensitiser, enabling its controlled release upon exposure to light. After 90 min of irradiation with visible light (λ ≥ 410 nm), over 80% of the drug was released, whereas under dark conditions no significant release was observed even after 72 h. In LN18 cells, irradiation reduced the IC_50_ value of Acr-Cbl nano from 54 to 15 μM, whilst for the photolysis product Acr-OH, the IC_50_ value decreased from 56 to 38 μM, indicating an additional contribution of the released chlorambucil to the system’s cytotoxicity. At the same time, the percentage of apoptotic cells increased following photoactivation of Acr-Cbl nano from 3.93 to 10.37 per cent, which the authors attributed to the combined action of ROS and released chlorambucil [15].

Temozolomide (TMZ) was used as a chemotherapeutic agent in both NCP [18], and OT@COF-RVG [32]. TMZ is an oral, monofunctional methylating agent from the imidazotetrazine group and a key component of chemotherapy used to treat GBM, particularly in combination with radiotherapy [71]. Under physiological conditions, TMZ is spontaneously converted into the active metabolite MTIC, and subsequently into the reactive methyldiazonium cation, which methylates DNA primarily at the N7 position of guanine and, to a lesser extent, at the N3 position of adenine and the O6 position of guanine. Although O6-methylguanine accounts for only a small proportion of the adducts formed, it is responsible for a significant proportion of TMZ’s cytotoxicity through mispairing with thymine and activation of the mismatch repair (MMR) system [72]. One of the main mechanisms of resistance to TMZ is the activity of O6-methylguanine-DNA methyltransferase (MGMT), which removes a methyl group from O6-methylguanine. Methylation of the MGMT promoter is associated with reduced expression of this enzyme and a greater benefit from TMZ treatment in patients with GBM [73]. The NCP utilised the controlled release of TMZ from the nanocomposite. In PBS at pH 7.4, only approximately 15 per cent of the drug had been released after 120 h, whereas in a pH 6.9 environment, laser irradiation accelerated its release to 87 per cent within 40 min. Combining the NCP with laser irradiation also increased cytotoxicity against C6 cells, indicating a synergistic effect between TMZ chemotherapy and the photo-induced effect [18]. In OT@COF-RVG, TMZ was encapsulated together with the O-GlcNAc transferase inhibitor OSMI-4, with the TMZ loading level at 62.1 per cent. OSMI-4 reduced the level of O-GlcNAcylation and significantly enhanced the cytotoxicity of TMZ against GBM cells, acting as a chemotherapeutic sensitiser. At the same time, the inhibition of O-GlcNAcylation promoted the lysosomal degradation of PD-L1 and reduced its expression, extending the platform’s action to include an immunomodulatory component associated with PD-L1 checkpoint blockade. In the GL261 intracranial glioma model, the entire OT@COF-RVG formulation inhibited tumour growth, increased apoptosis rates and prolonged survival; the authors indicated that the inhibition of O-GlcNAcylation by OSMI-4 may enhance the efficacy of TMZ in vivo [32].

In the BTP-7/BR2-ICG/MTO-LP regimen, mitoxantrone (MTO) was used as the chemotherapeutic agent [30]. MTO is a synthetic anthracenedione anticancer drug whose primary mechanism of action involves intercalation with DNA and stabilisation of the topoisomerase II–DNA complex, leading to the accumulation of DNA breaks and cell death [74,75]. In GBM, mitoxantrone is not part of the standard treatment regimen; however, its efficacy has been clinically evaluated primarily in the local treatment of recurrent tumours. In a study of 22 patients with recurrent GBM, in whom locally administered MTO was combined with systemic TMZ, the median progression-free survival was 7 months and the median overall survival was 11 months, with relatively good tolerability of the treatment [76]. In BTP-7/BR2-ICG/MTO-LP, mitoxantrone was co-encapsulated with ICG, with an MTO encapsulation efficiency of approximately 96 ± 1% (mean ± SD, n = 3). The drug was released from the liposomes gradually, without a sharp initial surge; in a medium containing 10% FBS, less than 40% of MTO was released after 48 h. In C6 cells, the IC_50_ of the doubly modified formulation was approximately 101 ± 1 ng/mL, compared with 290 ± 2 ng/mL for free MTO and 210 ± 1 ng/mL for PEG-ICG/MTO-LP (mean ± SD, n = 3), indicating increased cytotoxicity of the dual-ligand liposomal formulation. Additional activation of ICG by NIR light enhanced ROS generation and the photothermal effect, enabling the combination of MTO’s action with photo-induced therapy. However, the anticancer efficacy of this formulation has not been assessed in vivo; therefore, the observed increase in MTO activity requires confirmation in animal models [30].

In Cela@Lip-IRH, celastrol was used as an anti-cancer component [23]. Celastrol is a natural pentacyclic quinonometide triterpenoid with pleiotropic anti-tumour activity. One of its mechanisms of action involves disrupting mitochondrial function; it has been shown to inhibit complex I of the respiratory chain, leading to increased ROS production, loss of mitochondrial membrane potential and activation of the cytochrome C- and caspase-dependent apoptotic pathway [77]. In GBM models, it has been shown in vitro that it inhibits the proliferation, migration and invasion of cancer cells, and may disrupt mitochondrial homeostasis and increase the production of mitochondrial ROS [78,79]. In the U87 orthotopic model, celastrol also inhibited tumour growth, angiogenesis and vascular mimicry, which was attributed to the inhibition of the PI3K/Akt/mTOR pathway [78]. However, its therapeutic applications are limited by its poor water solubility, low bioavailability and off-target toxicity, which justifies the use of nanocarriers [80]. In Cela@Lip-IRH, the pH-dependent release of celastrol was utilised, promoting its availability in the slightly alkaline environment of the mitochondria. At pH 8.0, following NIR irradiation, almost 90% of Cela was released within 48 h, whilst at pH 6.8 and 5.0, these values were approximately 67% and 17%, respectively. In C6 cells, the IC_50_ value of Cela@Lip-IRH decreased following NIR irradiation from 0.53 to 0.15 μg/mL, and the combination index for Cela and photoactivated IR780 was 0.106, confirming strong chemo-PDT synergy. This effect was accompanied by the activation of the mitochondrial apoptotic pathway, including a decrease in mitochondrial membrane potential, an increase in Bax, the release of cytochrome C, and the activation of caspases-9 and -3. Its efficacy was also confirmed in a subcutaneous C6 glioma model [23].

## 5. Delivery and Targeting Strategies for Glioblastoma

The effective delivery of drugs to GBM poses a particular challenge due to the presence of the blood–brain barrier (BBB) [81], heterogeneous vascular permeability of the tumour [82], the diffuse and infiltrating nature of tumour growth [83] and a dense extracellular matrix that limits the penetration of drugs into the tumour tissue [84]. The nanoplatforms analysed employ strategies of varying degrees of sophistication—ranging from passive internalisation by cancer cells, through targeting of specific receptors and organelles, to active facilitation of transport across the BBB. This is particularly important in combination therapy involving PDT and chemotherapy, as the co-delivery of a photosensitiser and a chemotherapeutic agent in a single nanocarrier may improve their accumulation in the tumour, their bioavailability and the temporal coordination of their action [14], and, in some cases, also to a specific subcellular compartment [85].

The simplest delivery strategy was employed in Acr-Cbl nano, which contains neither a GBM-specific ligand nor a component designed for active transport across the BBB. However, in vitro studies demonstrated time-dependent uptake of the nanoparticles by LN18 cells [15]. In the case of nanoparticles without ligands, internalisation depends primarily on their physicochemical properties and may occur via non-specific endocytic mechanisms [86]. Whilst this delivery method may enable effective cellular uptake in vitro, it does not ensure selectivity for GBM or the ability to cross the BBB. As Acr-Cbl nano has only been evaluated in vitro, its bioavailability in the brain, transport across the BBB and accumulation in the tumour remain unknown [15].

In NCP (ZnO-Cys-Flx-TMZ-MB), a more targeted delivery strategy was employed through the incorporation of fluoxetine (Flx), which the authors used as a component to target AMPA receptors (AMPARs) expressed by glioma cells [18]. The rationale behind this strategy is the high expression of AMPARs in high-grade gliomas, including the overexpression of the GluR1/GluA1 subunit and Ca^2+^-permeable AMPARs in GBM [87,88]. Elevated expression of the GluR1 subunit of the AMPA receptor was observed in both GBM cell lines and patient-derived glioma samples, compared with astrocytes and normal brain tissue; furthermore, GluR1 levels increased with the grade of glioma malignancy, and in GBM cells the receptor was mainly localised on the cell surface [89]. Furthermore, AMPARs also play a role in regulating the proliferation, migration and invasion of glioma cells [87,88], and fluoxetine inhibits the growth of glioblastoma multiforme by inducing calcium-dependent apoptosis, which is mediated by the AMPAR [89]. The NCP study assessed the effect of fluoxetine on cellular uptake using flow cytometry; however, no direct validation of the specificity of the AMPAR-dependent mechanism was carried out, e.g., by blocking the receptor or by comparison with cells exhibiting low AMPAR expression. This strategy should therefore be regarded as a receptor-targeted delivery concept, the selectivity of which requires further confirmation. Nor was the transport of the NCP across the BBB or its in vivo biodistribution assessed; consequently, the ability of the entire nanocomposite to reach a brain tumour following systemic administration remains undetermined [18].

A more advanced delivery strategy was employed in Cela@Lip-IRH, in which IR780 acts not only as a photosensitiser but also contributes to preferential internalisation of the nanoplatform by tumour cells and, after cellular uptake, to its subcellular localisation within mitochondria [23]. The preferential tumour-cell accumulation of IR780 has been associated with transport mediated by organic anion-transporting polypeptides (OATPs), whereas its lipophilic cationic nature promotes mitochondrial accumulation due to the negative mitochondrial membrane potential [90,91,92]. In the nanoplatform under investigation, modification of IR780 significantly increased the uptake of nanoparticles by C6 cells. After 12 h of treatment and NIR irradiation, flow-cytometric analysis showed that the fraction of C6 cells exhibiting nanoparticle-associated RITC -labelled fluorescence was 98.6% for Lip-IRH, compared with 54.2% for unmodified Lip nanoparticles. Furthermore, confocal microscopy showed greater spatial overlap between the fluorescence of RITC-labelled Lip-IRH nanoparticles and MitoTracker-stained mitochondria, indicating enhanced mitochondrial localisation of the nanoplatform [23]. This subcellular targeting is of particular importance for PDT, as the short range of action of ROS means that localising the photosensitiser near sensitive organelles can enhance the effectiveness of the therapy [93,94]. IR780 also enhanced the ability of the nanoparticles to penetrate three-dimensional C6 spheroids, and NIR irradiation further amplified this effect. Cela@Lip-IRH exhibited the strongest fluorescence signal at all analysed depths within the spheroids, indicating improved penetration within the 3D tumour model. In an in vivo study conducted in a subcutaneous C6 model, Lip-IRH showed greater accumulation in tumour tissue than the unmodified carrier and persisted in the tumour for up to 96 h. These results confirm the beneficial effect of the IR780 modification on the nanoplatform’s penetration and retention within the tumour; however, the use of a subcutaneous model does not allow for an assessment of its ability to cross the BBB or to be delivered to intracranial GBM [23].

A much more sophisticated targeting strategy was employed in BTP-7/BR2-ICG/MTO-LP, the surface of which was functionalised with two peptides—BTP-7 and BR2 [30]. BTP-7 is a peptide that targets the deglycosylated isoform of brevican (dg-Bcan). Brevican (Bcan) is a CNS-specific extracellular matrix glycoprotein whose expression is elevated in GBM [95,96]. Of particular significance is its non-glycosylated isoform, dg-Bcan, which is selectively detected in high-grade human gliomas and has been proposed as a tumour-specific marker [97,98,99]. BTP-7 was selected as a ligand exhibiting specific affinity for recombinant dg-Bcan [96,100,101]. Subsequent studies have shown that its use may also increase the penetration of carriers through the blood–brain barrier, and that BTP-7-modified liposomes retain their ability to specifically recognise glioma cells once they have crossed the barrier [102,103]. To complement this recognition and transport function with effective penetration of the cell membrane, the BR2 peptide was also incorporated into the nanoplatform under investigation. BR2 interacts with membrane gangliosides, which facilitates its entry into cells via a lipid-dependent process [104,105]. Previous studies showed preferential internalisation of BR2 in selected malignant cell lines compared with non-malignant cells, with approximately fourfold higher transduction efficiency reported in the malignant cells tested [106]. Similar penetration efficiency was observed across the different malignant cell lines examined [105]. Despite this preferential uptake observed in the tested cell lines, BR2 is not a GBM-specific targeting ligand. For this reason, in BTP-7/BR2-ICG/MTO-LP, it was combined with BTP-7, resulting in a complementary system in which BTP-7 is responsible for more specific recognition of glioma, whilst BR2 facilitates membrane penetration and internalisation of the nanocarrier. The efficacy of dual functionalisation was confirmed in cellular uptake studies and a BBB model. In C6 cells, the uptake of BTP-7/BR2-CO6-LP was approximately 1.4-, 1.8- and 2.0-fold higher, respectively, than that of BR2-CO6-LP, BTP-7-CO6-LP and unmodified CO6-LP. In the Transwell model, following passage through the bEnd.3 layer, uptake by C6 cells was approximately 2.3-, 2.4- and 4.7-fold higher, respectively, than for the corresponding single-ligand formulations and PEG-CO6-LP. The doubly modified liposomes also exhibited the deepest penetration of C6 spheroids, indicating a complementary action of BTP-7 and BR2 in transport across the BBB model, cellular uptake and tumour model penetration. However, the platform’s ability to cross the BBB and deliver targeted therapy was not assessed in vivo [30].

In OT@COF-RVG, active targeting is based on the RVG-29 peptide [32]. RVG is a glycoprotein of the rabies virus responsible for its neurotropism. Its peptide derivatives, including RVG-29, are used as ligands to direct carriers to the CNS, presumably mainly through interaction with nicotinic acetylcholine receptors (nAChR), although other molecules, such as NCAM, may also be involved. The nAChR-dependent mechanism forms the basis for the use of RVG-29 in the delivery of drugs to the brain [107]. GBM cells express several subtypes of nAChR, and transcriptomic analyses have shown that the expression of some of these is elevated compared with non-tumour tissue [108,109]. In the nanoplatform under investigation, modification of RVG-29 significantly increased the internalisation of COF-RVG by U87MG and U251 cells compared with unmodified COF_TFP-DABP_, and the fluorescence signal intensity increased with incubation time and nanoparticle concentration. The ability to cross the BBB was assessed in a Transwell assay using hCMEC/D3 or b.End3 endothelial cells and GBM cells in the lower chamber. After 24 h, COF-RVG exhibited a significantly higher fluorescence signal in U87MG or GL261 cells than unmodified COF, indicating a beneficial effect of RVG-29 on transport across the BBB model. The targeting effect was also confirmed in vivo, where increased accumulation of nanoparticles in the central part of the tumour was observed in tumour sections from mice treated with COF-RVG [32].

Importantly, enhanced transport across an in vitro Transwell BBB model should not be interpreted as direct evidence that a nanoplatform can cross the BBB and accumulate in an intracranial tumour in vivo. In the case of BTP-7/BR2-ICG/MTO-LP, the evidence for BBB transport is limited to in vitro endothelial models, and intracranial delivery has not been demonstrated [30]. Similarly, the increased tumour accumulation observed for Cela@Lip-IRH was demonstrated in a subcutaneous glioma model and therefore does not establish BBB penetration [23]. OT@COF-RVG provides stronger in vivo evidence, as increased nanoparticle accumulation was observed in an orthotopic glioma model [32]; however, tumour accumulation itself does not necessarily demonstrate transport across an intact BBB or confirm the specific mechanism responsible for brain entry. These distinctions are important when considering the translational potential of the targeting strategies described above.

## 6. Comparative Analysis of the Nanoplatforms

The five nanoplatforms analysed differ substantially in their structural complexity, photoactive components, irradiation requirements, chemotherapeutic agents, targeting strategies and level of preclinical validation. These differences should be taken into account when interpreting their reported therapeutic effects, as direct comparison of efficacy across the individual studies is limited by substantial heterogeneity in experimental design. The principal characteristics, advantages and limitations of the analysed systems are summarised in Table 1.

The comparison highlights a progressive increase in functional complexity across the analysed systems. Acr-Cbl nano represents the most structurally streamlined approach, integrating photosensitisation and light-triggered chemotherapy within a single molecular construct. Its simplicity may facilitate mechanistic interpretation, although the requirement for short-wavelength visible-light irradiation and the absence of active targeting or in vivo validation represent important limitations [15]. Acr-Cbl is therefore informative primarily as a proof-of-concept demonstrating molecular integration of chemotherapy and PDT, rather than as a directly translatable GBM platform. Its inclusion illustrates an important design lesson: successful integration of two therapeutic modalities is insufficient if the photophysical requirements are incompatible with the anatomical constraints of the target tumour. NCP introduces a multicomponent design combining methylene blue, temozolomide and a proposed AMPAR-targeting strategy combining methylene blue, temozolomide and a proposed AMPAR-targeting strategy. However, the specificity of this targeting mechanism was not directly demonstrated, and the system has so far been evaluated only in vitro [18].

Cela@Lip-IRH and BTP-7/BR2-ICG/MTO-LP use NIR-responsive cyanine dyes and incorporate more advanced targeting functions. In Cela@Lip-IRH, IR780 combines photodynamic activity with preferential mitochondrial localisation, while the formulation also enables pH-dependent release of celastrol. Its efficacy was demonstrated in vivo, although only in a subcutaneous C6 glioma model, which does not permit assessment of delivery across the intact or partially disrupted BBB in intracranial GBM [23]. BTP-7/BR2-ICG/MTO-LP uses a dual-peptide strategy intended to combine glioma recognition with enhanced cellular penetration and showed improved uptake in C6 cells, BBB models and tumour spheroids. Nevertheless, the absence of in vivo therapeutic evaluation limits conclusions regarding its systemic delivery and translational potential [30].

OT@COF-RVG represents the most multifunctional of the five systems, combining a photoactive COF scaffold, TMZ, the O-GlcNAc transferase inhibitor OSMI-4 and RVG-29-mediated targeting. In addition to predominantly Type I photodynamic activity, the system incorporates chemosensitisation and immunomodulatory effects and was the only platform evaluated in an orthotopic glioma model [32]. However, its increased structural and functional complexity may also create additional challenges for reproducibility, pharmacokinetic characterisation and comprehensive safety assessment.

Overall, the five nanoplatforms differ substantially in their design, targeting strategy, irradiation requirements and level of preclinical validation. These differences should be considered when interpreting the available data, particularly because the studies used heterogeneous experimental models and treatment conditions.

## 7. Assessment of the Effectiveness of Nanoplatforms

In the studies analysed, the efficacy of the nanoplatforms was assessed using various in vitro glioma models, whilst in vivo therapeutic studies were conducted only for Cela@Lip-IRH and OT@COF-RVG. The scope of validation for each system varied and included the assessment of cytotoxicity, mechanisms of cell death, three-dimensional models and, in selected cases, the immune response and anticancer activity in vivo. Acr-Cbl nano was evaluated in LN18 glioma cells. Photoactivation reduced the IC_50_ value from 54 to 15 μM, accompanied by increased ROS generation and enhanced apoptosis, indicating a synergistic effect between PDT and the released chlorambucil. However, the efficacy of the platform was not assessed in vivo [15]. In the case of NCP (ZnO-Cys-Flx-TMZ-MB), irradiation increased ROS generation and cytotoxicity against C6 cells, whilst simultaneously accelerating the release of TMZ; at pH 6.9, used as a model of the tumour microenvironment, drug release reached approximately 87% within 40 min. The results indicated a synergistic interaction between the chemotherapeutic and photo-induced effects; however, the assessment of efficacy was limited to in vitro conditions [18]. Cela@Lip-IRH demonstrated marked chemo-PDT synergy in C6 cells, with photoactivation reducing the IC_50_ of the formulation from 0.53 to 0.15 μg/mL. The therapy also induced immunogenic cell death and the maturation of dendritic cells. In a subcutaneous C6 glioma model, the combination of Cela@Lip-IRH with NIR led to 97.5% inhibition of tumour growth and increased recruitment of CD4^+^ and CD8^+^ lymphocytes [23]. However, caution is required when interpreting the observed increase in the recruitment of CD4^+^ and CD8^+^ lymphocytes, as the studies were conducted in BALB/c nude mice, which are characterised by impaired thymus development and a significant impairment of T-cell-mediated immunity [110]. BTP-7/BR2-ICG/MTO-LP was shown to exhibit increased cytotoxicity against C6 cells compared with free mitoxantrone; the IC_50_ of the formulation was approximately 101 ± 1 ng/mL, compared with 290 ± 2 ng/mL for MTO. This effect was accompanied by ROS generation and the photothermal activity of ICG. Despite the use of BBB models and three-dimensional spheroids, the therapeutic efficacy of the system was not assessed in vivo [30]. The most comprehensive validation was carried out for OT@COF-RVG. In vitro, OSMI-4 potentiated the effect of TMZ and reduced PD-L1 expression, whilst photoactivation increased ROS generation and induced immunogenic cell death. In the GL261-Luc orthotopic model, the platform inhibited tumour progression, reduced proliferation, increased apoptosis and prolonged animal survival. The anticancer effect was accompanied by the activation of an immune response, including increased presence of CD8^+^ lymphocytes and dendritic cell maturation, as well as reduced PD-L1 expression. Importantly, depletion of CD8^+^ lymphocytes attenuated the platform’s anti-tumour effect, indicating a functional role for the immune response in the efficacy of the therapy [32]. Overall, the results suggest that combining phototherapy with chemotherapy in nanoplatforms may enhance anticancer activity through the combined effects of direct cytotoxicity, ROS generation and the action of cytotoxic drugs, and, in more complex systems, also through the modulation of the immune response. However, a direct comparison of the efficacy of individual platforms remains limited by differences in the models used, irradiation conditions and treatment regimens. Of the five systems analysed, only two were evaluated therapeutically in vivo, and only OT@COF-RVG was tested in an orthotopic glioma model, which significantly limits the possibility of drawing direct comparative conclusions. An additional limitation concerns the biological endpoints used in several studies. MTT- and CCK-8-derived changes were considered only short-term metabolic readouts and were not treated as evidence of durable tumour-cell eradication or therapeutic efficacy. In particular, such assays do not establish loss of clonogenic capacity [111,112]. Consequently, platforms evaluated predominantly using metabolic assays should be regarded as providing mechanistic or screening-level evidence rather than therapeutically predictive evidence (Table 2).

**Table 2:** In vitro and in vivo evaluation of photoactive nanoplatforms for glioblastoma therapy.

Nanoplatform/Photoactive Component	In Vitro—Model	In Vitro—Exposure/Assessment Time and Irradiation	In Vitro—Main Effect	In Vivo—Tumour Model/Treatment Initiation	In Vivo—Administration and Regimen	In Vivo—Irradiation	In Vivo—Main Therapeutic Outcome	Ref.
Acr-Cbl nano/acridine photocage–chlorambucil	LN18 glioblastoma cells; normal SVG P12 astrocytes	MTT-derived IC_50_ assessed after 48 h of formulation exposure; irradiated groups: visible light, λ ≥ 410 nm, 15 min	MTT-derived IC_50_: 54 µM (dark) vs. 15 µM (light); apoptosis increased from 3.9% to 10.4%; MTT metabolic activity in SVG P12 cells remained >80% of control up to 30 µM before photolysis	Not evaluated	—	—	—	[15]
Pt-NPs/Pt(II) complex	Kr158 glioblastoma cells	IC_50_ assessed 24 h post-treatment; irradiated groups: 390 nm, 1 h	MTT-derived IC_50_: 78 µM (dark) vs. 0.69 µM (light); phototherapeutic index = 114	Not evaluated	—	—	—	[113]
Pol(CuI-ICG)/CuI-ICG nanoparticles	CT26 colorectal cancer cells	Pol(CuI-ICG); X-ray 0–4 Gy; main mechanistic experiments at 10 µg Cu/mL + 2 Gy	Concentration-dependent cytotoxicity enhanced by X-ray; LC_50_ 5.2 µg Cu/mL at 2 Gy; hemolysis 0.5% at 20 µg Cu/mL	Subcutaneous CT26 tumour model; treatment initiated on post-implantation day 7	Tail-vein injection, 1.25 mg Cu/kg; irradiation 12 h post-injection	X-ray, 2 Gy	87.7% tumour volume reduction; stable body weight and normal behaviour; no detectable systemic toxicity	[114]
OT@COF-RVG/COF loaded with OSMI-4 and TMZ	U87MG and U251 GBM cells	COF*_TFP_*_−_*_DABP_* 30 µg/mL for carrier biocompatibility; white light 26 mW/cm^2^ for PDT experiments	CCK-8-derived metabolic activity remained >80% of control at 30 µg/mL COF; light activation increased ROS generation and cytotoxic effects.	Intracranial GL261-luc GBM model in C57 mice; treatment initiated 7 days after tumour implantation	Nanoparticle treatment once daily for 3 consecutive days; administration route not specified in the main therapeutic description	White light, 26 mW/cm^2^	Reduced tumour volume; ↓Ki-67; ↑cleaved caspase-3 and TUNEL positivity; minimal body-weight fluctuations; improved survival	[32]
bNe@AIE/Cas9-CD73/NDA-DPE AIEgen + CRISPR/Cas9-CD73	GL261 glioblastoma cells; normal BV2 microglia; isolated neutrophils	AIE dots tested up to 100 µg/mL in the dark; NIR laser 808 nm, 0.5 W/cm^2^, 5 min	Cell viability remained >80% at 100 µg/mL in all tested cell types under dark conditions; irradiation induced marked ROS production and apoptosis	Orthotopic GL261-Luc GBM model in C57BL/6J mice	Intravenous administration; phototherapy performed 48 h after injection	NIR laser, 808 nm, 0.5 W/cm^2^, 5 min	Strong inhibition of GBM progression and tumour reduction; significantly prolonged survival; immune activation; low systemic toxicity	[115]

Note: MTT-derived outcomes reflect cellular metabolic activity and should not be interpreted as direct measures of cell survival. Note: The reported 24 and 48 h intervals refer to in vitro exposure or assessment times used in the original studies and should not be interpreted as clinically applicable treatment durations. Note: Irradiation at 390 nm and the short-wavelength component of the broadband λ ≥ 410 nm irradiation used for Acr-Cbl nano are subject to strong tissue attenuation and therefore have limited relevance for direct activation of deeply located intracranial tumours. Longer wavelengths used by several other platforms, including 655 and 808 nm, provide comparatively greater tissue penetration, although light delivery remains a fundamental limitation of PDT in GBM [37].

## 8. Preliminary Safety and Biocompatibility Findings

The available studies provided only limited short-term safety and biocompatibility data for the analysed nanoplatforms, and the scope of these assessments varied considerably. Acr-Cbl nano exhibited low cytotoxicity towards SVG P12 cells under non-photoactivated conditions, with MTT-derived metabolic activity remaining >80% of control up to a concentration of 30 μM [15]. No comprehensive safety assessment has been carried out for BTP-7/BR2-ICG/MTO-LP; in particular, no in vivo systemic toxicity studies [30]. Cela@Lip-IRH was characterised by a low degree of haemolysis and did not cause any significant histopathological changes in the major organs [23]. Similarly, OT@COF-RVG did not cause any significant histopathological changes or abnormalities in the haematological and biochemical parameters examined, and furthermore showed a low degree of haemolysis [32]. Taken together, the available results indicate favourable short-term biocompatibility of the selected systems; however, the limited scope of the studies and the lack of data on long-term safety call for further assessment.

From a translational perspective, the available safety data remain insufficient, particularly for structurally complex inorganic and COF-based nanoplatforms. Short-term biocompatibility findings do not establish the long-term fate of these materials after systemic administration. Further studies should therefore address biodistribution, pharmacokinetics, degradation and clearance, as well as the possibility of prolonged accumulation in major organs or the CNS. For brain-directed nanoplatforms, conventional systemic toxicity assessment should additionally be complemented by evaluation of potential neurotoxicity and neuroinflammatory effects [116,117,118]. These issues are particularly important before the clinical translation of non-biodegradable or slowly degradable nanomaterials.

## 9. Lessons Learned and Translational Priorities

Taken together, the studies reviewed here suggest that increasing the structural complexity of a chemo-PDT nanoplatform does not necessarily increase its translational relevance. The principal lesson is that future systems should be designed around the specific constraints of intracranial GBM from the outset, rather than optimised primarily for performance in cell culture. This requires simultaneous consideration of clinically feasible light delivery, activity under hypoxic conditions, effective exposure of intracranial tumour tissue, and biologically meaningful measures of treatment efficacy. In this context, favourable cellular uptake, metabolic-assay responses or transport across an in vitro BBB model should be regarded as preliminary evidence rather than predictors of therapeutic success.

The available studies also indicate that future progress may require more than incremental optimisation of existing nanocarriers. Greater emphasis should be placed on approaches that directly address the physical limitations of PDT in the brain, including integration with interstitial or intracavitary light delivery, photochemical mechanisms less dependent on oxygen availability, and alternative excitation strategies capable of reaching deeper tissue. Such approaches remain experimental, but they provide a more rational direction for future development than further refinement of platforms whose irradiation requirements or experimental validation are poorly aligned with the clinical characteristics of GBM.

## 10. Conclusions

The available evidence demonstrates the technical feasibility of integrating chemotherapy and PDT within nanoparticle-based systems for glioma treatment, but does not yet establish their translational readiness. Of the five chemo-PDT platforms identified in the recent literature, three were evaluated only in vitro, only two underwent therapeutic testing in vivo, and only one was investigated in an orthotopic glioma model. Moreover, several reported effects were based on short-term metabolic assays or irradiation conditions with limited relevance to deep intracranial disease.

The principal value of the current studies therefore lies in the design lessons they provide rather than in evidence of an emerging clinical treatment. Future development should prioritise clinically feasible light delivery, photochemistry compatible with the hypoxic GBM microenvironment, demonstrable exposure of intracranial tumour tissue, and therapeutic endpoints that reflect durable tumour control. Whether nanoparticle-mediated chemo-PDT can ultimately improve clinical outcomes in GBM remains unresolved.

## Figures and Tables

**Table 1:** Comparative characteristics of chemo-photodynamic nanoplatforms investigated for glioblastoma treatment.

Nanoplatform	Photoactive Component/Irradiation Conditions	Chemotherapeutic Component	Targeting/Delivery Strategy	Experimental Validation	Principal Advantages	Main Limitations
Acr-Cbl nano	π-extended acridine photocage; 125 W medium-pressure Hg lamp with 1 M NaNO_2_ UV cut-off filter; λ ≥ 410 nm	Chlorambucil	No active GBM-specific targeting; passive cellular uptake	LN18 glioblastoma cells; in vitro only	Simple single-component architecture integrating light-triggered chlorambucil release with photodynamic ^1^O_2_ generation	Requirement for short-wavelength visible light; no GBM-specific targeting; BBB transport, biodistribution and in vivo efficacy not evaluated [15]
NCP (ZnO-Cys-Flx-TMZ-MB)	Methylene blue; 655 nm	Temozolomide	Fluoxetine-based AMPAR-targeting concept	C6 glioma cells; in vitro only	Integration of TMZ with photoinduced ROS generation; irradiation promoted drug release; receptor-targeting concept incorporated into the nanocomposite	Specificity of AMPAR-dependent targeting was not directly validated; BBB transport, biodistribution and in vivo efficacy were not assessed [18]
Cela@Lip-IRH	IR780; NIR laser, 808 nm	Celastrol	IR780-mediated preferential tumour-cell uptake and mitochondrial targeting	C6 cells, 3D spheroids and subcutaneous C6 glioma model	NIR-responsive system; mitochondrial targeting; pH-dependent celastrol release; chemo-PDT synergy and in vivo antitumour activity demonstrated	In vivo efficacy evaluated only in a subcutaneous model; delivery across the BBB and therapeutic efficacy in an intracranial GBM model remain unconfirmed [23]
BTP-7/BR2-ICG/MTO-LP	Indocyanine green; NIR laser, 808 nm	Mitoxantrone	Dual functionalisation with BTP-7 for glioma recognition and BR2 for enhanced cellular penetration	C6 cells, BBB Transwell model and 3D spheroids; no in vivo therapeutic evaluation	Dual-targeting strategy; NIR activation; combined photodynamic and photothermal activity; enhanced cellular uptake and penetration in BBB and spheroid models	BBB transport demonstrated only in vitro; therapeutic efficacy, biodistribution and systemic safety not validated in vivo [30]
OT@COF-RVG	COF(TFP-DABP); white light, 26 mW cm^−2^	Temozolomide with OSMI-4 as a chemosensitising and immunomodulatory adjunct	RVG-29-mediated CNS/GBM targeting	U87MG and U251 cells, BBB models and orthotopic GL261-Luc glioma model	Type I-dominant photodynamic activity; integration of TMZ sensitisation, immunomodulation and targeting; evaluation in an orthotopic glioma model	Structurally and functionally complex system; long-term safety, pharmacokinetics and biodistribution remain insufficiently characterised [32]

## Data Availability

No original experimental data or new datasets were generated in this review. All data and information discussed were derived from previously published studies cited in the manuscript.
